# *Melipona scutellaris* Geopropolis: Chemical Composition and Bioactivity

**DOI:** 10.3390/microorganisms11112779

**Published:** 2023-11-15

**Authors:** Sónia Coutinho, Vanessa Matos, Natália Seixas, Hellen Rodrigues, Vanessa B. Paula, Lais Freitas, Teresa Dias, Francisco de Assis Ribeiro Santos, Luís G. Dias, Letícia M. Estevinho

**Affiliations:** 1 Instituto Politécnico de Bragança, 5300-252 Bragança, Portugal; sonia_coutinho25@hotmail.com (S.C.); lais.sene.freitas@hotmail.com (L.F.); 2 Programa de Pós-Graduação em Botânica, Universidade Estadual de Feira de Santana, Avenida Transnordestina, s/n, Novo Horizonte, Feira de Santana 44036-900, BA, Brazilfasantos@uefs.br (F.d.A.R.S.); 3 Centro de Investigação de Montanha (CIMO), Instituto Politécnico de Bragança, 5300-252 Bragança, Portugaltdias@ipb.pt (T.D.); ldias@ipb.pt (L.G.D.); 4 Laboratório para a Sustentabilidade e Tecnologia em Regiões de Montanha, Instituto Politécnico de Bragança, Campus de Santa Apolónia, 5300-253 Bragança, Portugal

**Keywords:** beehive products, beehive products, geopropolis, natural products

## Abstract

Geopropolis has been used in traditional medicine for centuries. In this study, the botanical origin, physicochemical profile, and biological activities of geopropolis from *Melipona scutellaris* harvested during rainy and dry seasons were investigated. Palynological analysis identified over 50 pollen types, with *Schinus terebinthifolius* and *Cecropia* being the predominant types. The analytical results were in line with those reported in the literature. Rainy-season geopropolis exhibited higher total phenol and flavonoid content (determined using High Performance Liquid Chromatography—25.13% and 3.92%, respectively) compared to the dry season (19.30% and 2.09%); the major peaks (naringin, gallic acid, and catechin) were similar among samples. Antioxidant capacity was assessed via DPPH, reducing power, and β-carotene/linoleic acid discoloration assays. Rainy-season samples displayed superior antioxidant activity across methods. Antimicrobial effects were determined using microdilution, while the impact on the cholinesterase enzyme was quantified using 5-thio-2-nitrobenzoic acid accumulation. Anti-inflammatory and antimutagenic activities were assessed through hyaluronidase enzyme inhibition and by utilizing *Saccharomyces cerevisiae* ATCC-20113 cells. Both samples exhibited anti-inflammatory and antimutagenic properties. Moreover, a significant inhibition of acetylcholinesterase was observed, with IC50 values of 0.35 µg/mL during the rainy season and 0.28 µg/mL during the dry season. Additionally, the geopropolis displayed antimicrobial activity, particularly against *Staphylococcus aureus*. These findings suggest the therapeutic potential of *M. scutellaris* geopropolis in the context of inflammatory, oxidative, and infectious diseases.

## 1. Introduction

In Brazil, geopropolis has been used as a traditional medicine for centuries, being used to treat a variety of ailments including respiratory and gastrointestinal illnesses. Propolis and geopropolis are both resins collected by bees for hive construction and protection. While they share similarities, they are distinct in composition. Propolis, produced by honeybees from the *Apis* family, is a mixture of tree resins, wax, enzymes, salivary secretions, and other organic ingredients. On the other hand, geopropolis is created by stingless bees of the *Melipona* family, which mix propolis with clay or soil. As a result, geopropolis has a powdery texture, an earthy aroma, and a higher mineral content [1]. The chemical composition of propolis and geopropolis is highly complex and influenced by various factors, including geographic origin, the available plant sources for the bees, bee species, and soil type [1,2]. Brazil, being a megadiverse country with multiple geomorphological and climatic conditions, harbors more than 200 species, with around 20% of them being endemic [3]. According to Turco et al. [4], different *Meliponinis* species exhibit selective foraging behaviors, with *M. quadrifasciata* collecting resins from a wider range of plants and *Tetragonisca angustula* being more specific in their choice of vegetal sources. These variables contribute to the existence of numerous types of geopropolis and propolis, each with a chemical composition that is both qualitatively and quantitatively unique [4,5,6]. These substances typically contain phenylpropanoids, flavonoids, phenolic acids, hydrolysable tannins, triterpenes, saponins, alkaloids, lipids, and other compounds [1,2,4,6,7,8], which are associated with biological activities such as antioxidant [6,7,8,9], anti-inflammatory [6,9], antimicrobial [6,9] and anticancer [6,10] activities. Geopropolis is undoubtedly a great source of molecules with different biological properties, being used in traditional medicine of different peoples, and there are possibilities for new discoveries, with the search for phenolic compounds being the most promising [11].

Despite the correlation between bioactive compounds and their plant sources, there is a lack of information regarding the specific plants used by bees for geopropolis production in Brazil. Furthermore, there has been no study on the effect of the season on the quality and bioactivity of geopropolis. The main objective of this work was to evaluate the effect of climatic conditions on the pollen profile, physicochemical characteristics, phenolic profile, biological activities, and mutagenic activity.

## 2. Materials and Methods

In this study, several samples of geopropolis produced by *Melipona scutellaris* were collected, some in the rainy season (months between April and September) and others in the dry season (months between October and March). Geopropolis was collected at different times of the year, as these temporal variations can exert an influence on its attributes, thereby impacting the therapeutic qualities of geopropolis. The chemical compounds of geopropolis depend on the plant sources, geographical zone, season, and bee species. As the amount of geopropolis produced by stingless bees is very small, for the realization of the work, two composite samples—one consisting of the samples from the dry season and the other from the rainy season—were made. The samples were obtained from the meliponary of Fazenda São Lucas (12°01′786″ S and 38°04′366″ W), located in a region with predominance of secondary Atlantic Forest, in the municipality of Entre Rios. This municipality is located on the BR-110 highway between Salvador and Alagoinhas in the state of Bahia, Northeast Brazil. The state of Bahia has about 564,733.081 km^2^, with 417 municipalities, occupying approximately 36.4% of the Northeast region [12].

### 2.1. Reagents

Methanol and ethanol were obtained from Pronolab (Lisbon, Portugal). Culture media (Mueller Hinton) was obtained from Sigma-Aldrich (St. Louis, MO, USA). TTC solution (2,3,4-triphenyl-2H-tetrazolium chloride) was from Fluka (Buchs, Switzerland). Folin–Ciocalteau, DPPH, chloroform, sodium carbonate, calcium chloride, quercetin, gentamicin, amphoterecin, linoleic acid, glacial acetic acid, hydrochloric acid (HCL), acetate buffer, and the other reagents were obtained from Sigma Chemical Co. (St. Louis, MO, USA). The methanol used in the HPLC analysis was HiPerSolv CHROMANORM, 99.8% pure (VWR BDH Prolabo, Lutterworth, UK), and the formic acid came from Merck (Darmstadt, Germany). The standards for HPLC analysis, as the gallic acid (≥99%), protocatechuic acid (99.63%), (+)-catechin (≥98%), (−)-epicatechin (≥97%), vanillic acid (≥97%), resorcinic acid (≥97%), chlorogenic acid (>95%), caffeic acid (≥98%), sirinic acid (≥98%), p-coumaric acid (≥98%), ferulic acid (≥99%), sinapic acid (≥99%), rutin hydrate (≥94%), quercetin (95%) kaempferol (≥98%), naringin (≥95%), naringenin (98%), and cinnamic acid (≥99%), were purchased from Sigma-Aldrich (Steinheim, Germany). All enzymes were purchased from Sigma-Aldrich (Sternheim, Germany).

Ultra-pure water (18 MΩcm) was obtained in a Mili-Q purification system (Millipore, Bedford, MA, USA).

### 2.2. Palynological Analysis

The samples were processed according to the methodology described in [13]. For the palynological census, a minimum of 500 pollen grains per geopropolis sample was established. The identification of the botanical affinity of the pollen types was performed according to the indications of [14]. The number of samples in which a given pollen type was present (distribution frequency) was also observed. According to [15], samples were categorized into the following frequency classes: very frequent—>50%; frequent—20–50%; infrequent—10–20%, and rare—<10%. The pollen grains were identified taxonomically with the aid of the slides deposited in the palynoteca of the Laboratory of Plant Micromorphology (UEFS), in which all the prepared slides were also deposited. Catalogs and other works were also used to assist in the identification of the botanical origins of the pollen types. After completion of the analysis, the main pollen types were photomicrographed under an optical microscope.

### 2.3. Physico-Chemical Analysis

As reported by [16], the following parameters were evaluated: moisture, pH, conductivity, ash, and wax content. All analyses were performed in triplicate.

The determination of moisture was ascertained using the standard method (AOAC Official Method 934.01). Five grams of geopropolis were dried in a mechanical convection oven at 105° for 1 h. After this time, it was removed and let to cool at room temperature and weight back. The water content was determined using an equation in which A1 = weigh of sample and A2 = weigh of sample dried: Moisture (%) = 100 × (A1 − A2)/A1.

The pH of geopropolis was measured with a combined pH glass electrode connected to pH-meter Basic 20 in a solution prepared with 10 g of geopropolis in 75 mL of methanol (NP 1309/1976), using methanol as control. Calibration was performed with three standard buffer solutions. 

Electrical conductivity of a geopropolis solution at 20% (*w*/*v*) (dry matter basis) in methanol was measured at 20 °C in a Crison 522 conductimeter. Results were expressed in milliSiemens per centimeter (mS/cm).

The method used in the experiments to determine the mineral content and other inorganic matter in geopropolis consisted of the desiccation of an amount of 5 g, for each geopropolis sample, in a platinum dish (AOAC Official Method 920.181). To do so, they were kept in the thermostat at 80 °C for 4 h, after which the samples underwent calcination at 550 °C in an electric laboratory furnace, SNOL 8.2/1100-1 (AB ‘‘Umega’’, Utena, Lithuania), to constant mass. Total ash content, expressed as the percentage of residue left after dry oxidation by weight (%), was calculated from the following equation, where m1 is the mass of dish and ash, m2 is the mass of platinum dish prior to calcination, and m0 is the mass of the propolis taken: Ash (%) = (m1 − m2/m0) × 100.

For wax determination, we weighed 250 g of each sample and added 750 mL of methanol. The mixture was placed in a freezer overnight (−20 °C). Afterwards, the solution was filtered to obtain the wax. The wax was expressed in percentage (W%) using the sample weigh (SW) and the wax weigh (WW). 

The equation used was W (%) = WW/SW × 100.

### 2.4. Preparation of Ethanolic Extracts

The geopropolis samples were frozen at −4 °C, crushed, and sieved to a homogeneous powder. Subsequently, 15 g of the samples was diluted in 240 mL of 70% ethanol. The mixture was stirred (200 rpm) for 24 h at room temperature, after which it was refrigerated for 12 h, filtered, rotoevaporated at 40 °C, and lyophilized [17]. The yields were 3.62 g (summer sample) and 4.05 g (winter sample).

### 2.5. Quantification of Total Phenols and Flavonoid Content

**(a)**  **Determination of total phenols**

Total phenolic compounds were determined according to the Folin–Ciocalteau colorimetric method [16]. A volume of 0.5 mL of the geopropolis extract (100 µg/mL) was mixed with 2.5 mL of Folin–Ciocalteau reagent and 2.0 mL of 14% sodium carbonate solution (Na_2_CO_3_). The mixture was incubated for 2 h in the dark at room temperature. After this period, the absorbance was read at 760 nm. The calibration curve was made using gallic acid (0.3–12 µg/mL) as standard. The results, obtained in triplicate, were expressed in percentage format (%).

**(b)**  **Determination of total flavonoid content**

The evaluation of total flavonoid content was performed as described by Castro et al. [18] with slight modifications. A quantity of 0.5 mL of extract (from 0.1 to 200 µg/mL) was diluted with 2.5 mL of ethanol solution of aluminium chloride hexahydrate 2% (AlCl_3_·6H_2_O). Then, after incubation for 1 h at room temperature, the absorbance of the mixture was read at 420 nm. Quercetin (0.3–18 µg/mL) was used as standard. The flavonoid content results, obtained in triplicate, were expressed in %.

### 2.6. Antioxidant Activity

The huge diversity of antioxidant compounds present in natural products makes it difficult to separate and study them individually. Indeed, individual antioxidant compounds do not necessarily reflect the total antioxidant capacity of the product, as there may be synergistic or antagonistic interactions between the different compounds present. The methods often used to determine in vitro antioxidant capacity can be broadly divided into two categories: hydrogen atom transfer (HAT) reaction-based assays and electron transfer (ET) reaction-based assays. In our study we used three methodologies to evaluate the antioxidant capacity of geopropolis extracts, two based on TE (DPPH radical scavenging activity assay and reducing power) and one based on HAT (β-carotene decolorization inhibition assay) [17,19].

**(a)**  **Scavenging of DPPH radicals**

In the DPPH (1,1-diphenyl-2-picrylhydrazyl) radical scavenging activity (RSA) assays, the methodology described by Moreira et al. [20] was followed. An aliquot of 0.3 mL of geopropolis extract (concentrations ranged from 0.1 to 175 µg/mL) was mixed with 2.7 mL of DPPH reagent (2.0 × 10^−4^ M). The mixture was left to stand in the dark for 60 min. After this time, the absorbance of the solutions, read at 517 nm using ascorbic acid, was used as the standard to establish the calibration curve. The IC50 was calculated as the percentage of decolorization of the DPPH solution, according to the following equation:

% IC50 = [(Abs_517_ − A_517_ S)/Abs_517_ DPPH] × 100

Here, Abs_517_ S-corresponds to the absorbance of the DPPH solution in the presence of different concentrations of extract and Abs_517_ DPPH to the absorbance of the blank (DPPH solution plus extraction solvent instead of extract solution).

**(b)**  **Reducing power**

This methodology was based on the protocol reported by Moreira et al. [20] (slightly modified). A volume of 0.25 mL (1 mg/mL) of the sample extract was mixed with 1.25 mL of phosphate buffer (0.2 M, pH 6.6) and 1.25 mL of 1% potassium ferricyanide. The mixture was placed in a bain Marie at 50 °C for 20 min. Then, after 1.25 mL of 10% trichloroacetic acid was added to this solution, it was centrifuged at 3000 rpm for 10 min. Subsequently, from the supernatant, 1.25 mL was removed and added to 1.25 mL of deionised water and 0.25 mL of 0.1% FeCl_3_. The absorbance was read at 700 nm and the results were noted. The concentration of extract that induced a 50% inhibition percentage (EC50) was calculated from the plot of absorbance (700 nm) as a function of extract concentration in the solution. BHA (Butylated hydroxyanisole) was used as a control.

**(c)**  **Discoloration of β-carotene/linoleic acid**

This assay was performed according to the method described by Ahn et al. [21] with some modifications.

Before starting the test, the chloroform solution of β-carotene was prepared (1 mg of β-carotene dissolved in 5 mL of chloroform). Then, 1 mL of this solution was mixed with 20 mg of linoleic acid and 200 mg of Tween 40. After extracting the chloroform using a rotary evaporator at 40 °C, 50 mL of oxygenated distilled water was slowly added to the mixture and the flask was shaken vigorously to form an emulsion. For this test, 250 µL of the emulsion was added to 30 µL of the geopropolis extract in a microplate. Absorbance measurements were taken at 492 nm immediately after adding the emulsion to the extract, as well as again after 120 min. The microplate was placed on a shaker at 50 °C. As a control, 30 μL of the solvent (80% ethanol) was incubated with 250 μL of the emulsion. Independent experiments were carried out in triplicate.

The inhibition of β-carotene decolorization was calculated using the following equation:

% Discoloration = [(Abs_470_ after 2 h/Abs_470_ initial) × 100]

### 2.7. Anti-Inflammatory Activity

Anti-inflammatory activity was assessed via inhibition of hyaluronidase enzyme, using the method described by Sahasrabudhe et al. [22]. A volume of 150 µL of bovine hyaluronidase enzyme (7900 units/mL) was dissolved in acetate buffer (pH 3.6). Subsequently, 25 mL of geopropolis extract (0.2–100 mg/mL) was added and the mixture was incubated for 30 min at 37 °C. Then, 50 µL of calcium chloride (12.5 mM) was added and incubated again under the same conditions. After this period, 250 µL of sodium hyaluronate (1.2 mg/mL) was added and the mixture was incubated again at 37 °C for 1.5 h. After this period, 50 mL of 0.4 M sodium hydroxide and 100 mL of 0.6 M sodium borate were added and placed in a water bath for 3 min. Finally, it was cooled on ice and 1.5 mL of PDMAB (p-dimethylaminobenzaldehyde) was added (4 g PDMAB dissolved in 50 mL of 10 M HCl and 350 mL of glacial acetic acid). The absorbance was measured at 585 nm (spectrophotometer: UV-VIS 3100PC). The assay was performed in triplicate. Epigallocatechin was used as control. The percentage of hyaluronidase enzyme inhibition was calculated according to the following equation:

Inhibition of the enzyme hyaluronidase (%) = ((Abs_600_ control Abs_600_ sample)/Abs_600_ control) × 100

### 2.8. Antimicrobial Activity

The biological material used in this work comprised strains of *Staphylococcus aureus* ATCC provided by the American Type Culture Collection 43300 (Gram-positive bacteria) and *Proteus mirabilis ATCC* 10231 (Gram-negative bacteria) and *Candida albicans* (yeast) ATCC 25933 from the collection (LGC Standards SLU, Barcelona) ATCC. Clinical isolates from Centro Hospitalar do Nordeste (Portugal), identified at Escola Superior Agrária do Instituto Politécnico de Bragança using molecular biology techniques, were also used. *S. aureus* ESA 321 was isolated from purulent exudates, *P. mirabilis* ESA 229 was isolated from an algalia (Foley probe), and *C. albicans* ESA 115 was isolated from sputum.

Mueller Hinton Broth (MHB, Sigma) and Mammalian Cell Culture Media (RPMI, Sigma) were employed to determine the minimum inhibitory concentration (MIC) and the minimum microbicidal concentration (MMC). The MIC value represents the lowest concentration of geopropolis extract capable of inhibiting microbial growth (including bacteria and yeast), and MBC, the lowest concentration of the geopropolis extract that prevents microbial growth and reduces initial viability by at least 99.9%. The methodology used closely followed that outlined in the work of Moaris et al. [19], with certain modifications. Geopropolis extract samples were dissolved in 50% DMSO and Mueller–Hinton medium for bacterial testing, and in 50% DMSO and Mammalian Cell Culture Media for yeast testing, resulting in a final concentration of 20 mg/mL.

In each well of the microplate, 100 µL of MHB and RPMI medium were placed for bacteria and yeasts, respectively. Then, 50 µL of the different concentrations of extract to be tested and 50 µL of cell suspensions (1 × 10^5^ for bacteria and 1 × 10^3^ for yeasts, grown in appropriate medium overnight) were introduced into the various wells. After being covered with sterile semi-permeable film, the microplates were incubated at 37 °C for 24 h. At the end of this time, the optical density was determined at 600 nm and 540 nm, respectively for yeasts and bacteria.

To determine the MIC, 20 µL samples from both the last well where color change was observed as well as from all wells that remained unchanged were inoculated onto plates with MHB for bacteria or RPMI for yeast and incubated at 37 °C for 24 or 48 h. The lowest concentration that did not result in growth after this subculture was considered the MIC. All assays were performed in triplicate.

### 2.9. Antimutagenic Activity

The antimutagenic capacity of geopropolis extracts was determined using yeast cells—D7 diploid strain of *Saccharomyces cerevisiae* (AmericanTypeCultureCollection, ATCC201137) [23]. Before starting the tests with geopropolis, the *Sacharomyces cerevisiae* colonies were evaluated for the frequency of spontaneous gene conversions at the tryptophan locus and reverse mutations at the isoleucine locus. Cultured cells with low spontaneous gene conversion and low reverse mutation frequency were inoculated into liquid medium at 28 °C until they reached stationary growth phase. The cultures were centrifuged and resuspended in sterile potassium phosphate buffer (0.1 M), pH 7.4, until a cell concentration of 2 × 10^8^ cells/mL was obtained. Subsequently, the mutagen ethyl methanesulphonate (EMS) (5 mg/mL) and the extract, evaluated at concentrations of 2.5 and 5.0 mg/mL, were added.

The mixture was incubated with stirring for 2 h at 37 °C. After this period, the cells were placed in a complete and selective incubator to determine yeast survival, tryptophan converters, and isoleucine reversers. The experiments were carried out in triplicate.

### 2.10. Inhibition of Acetylcholinesterase

The enzyme acetylcholinesterase converts acetylcholine into acetic acid and thiocoline. The latter compound reacts with 5,5′-dithiobis-2-nitrobenzoic acid (DTNB) with formation of 5-thio-2-nitrobenzoic acid. The accumulation of 5-thio-2-nitrobenzoic acid was assessed at 405 nm. The acetylcholinesterase inhibition was determined spectrophotometrically, as described by Mata et al. [24]. The 5,5-dithiobis-(2-nitrobenzoic acid) (15 mM, DTNB, Sigma Chemical, St. Louis, MO, USA) and the electroreflective acetylcholinesterase substrate (electric eel Type-VI-S, Sigma Chemical, St. Louis, MO, USA) were used. Acetylcholinesterase hydrolysis was assessed through the formation of 2-nitro-5-thiobenzoate anion (yellow) at 412 nm for 15 min. To determine the percentage of inhibition, the reaction speeds of the samples were compared with those of the blank (ethanol in 0.2 M of phosphate buffer, pH = 8), using the expression ((E − S)/E) × 100, where E is the enzyme activity without the extract and S is the activity of the sample. The IC50 values were estimated from the graph of the inhibition percentage as a function of the sample concentration. Eserine was used as a control.

### 2.11. Identification and Quantification of Compounds

Identification and quantification of phenolic compounds were performed according to the method previously described by Nastić et al. [25]. The detection of the phenolic profile of the extracts was obtained via high performance liquid chromatography with UV detection (HPLC/UV). The analyses were carried out at the Laboratory of the Materials Center of the University of Porto/CEMUP. Geopropolis samples were solubilized in HPLC grade methanol to a final concentration of 10 mg/mL, filtered through 0.2 µm Millipore nylon filters, and stored in 2.5 mL amber glass vials.

The quantification and separation of phenolic compounds by HPLC-UV was performed based on the procedure described by Rubilar et al. [26], with some modifications. The chromatograph used was a Shimadzu (LabSolutions Software, version 5.3) with column oven (CTO-10AS vp), injection system (LC-20AD), auto sampler (SIL-20A ht), and photodiode array detector (SPD-M20A), equipped with a Phenomenex Gemini C18 reverse-phase column (250 × 4.6 mm; 5 μm). The extracts were filtered through 0.22 µm nylon filters (Millipore) and a volume of 20 µL was injected. The mobile phase consisted of water (solvent A) and methanol (solvent B), both acidified with 0.1% (*v*/*v*) formic acid, at a flow rate of 1.0 mL/min in gradient mode as follows: 0 min, 15% of B in A; 20 min, 30% of B in A; 40 min, 45% of B in A; 45 min, 50% of B in A; 50 min, 55% of B in A; 65 min, 70% of B in A; 75 min, 100% of B; and 100% of B. This was maintained (75–80 min); the mobile phase composition returned to the initial conditions within the next ten minutes and was kept for another 10 min for stabilization before the next injection. The eluents were filtered through a nylon filter (0.20 µm pore size, Supelco, Bellefonte, PA, USA) and degassed. During the analyses, the column temperature was maintained at 25 °C. Detection of phenolic compounds was performed via scanning between 190 and 600 nm, and quantification was performed at 280 nm for monomeric flavan-3-ols (catechin and epicatechin), hydroxybenzoic acids (gallic, vanillic, protocatechuic, syringic, and β-resorbic acids), naringin, naringenin, and cinnamic acid; at 320 nm for hydroxycinnamic acids (caffeic, chlorogenic, p-coumaric, ferulic, and sinapic); and at 360 nm for rutin, quercetin, and kaempferol. The analytes in each extract were identified through comparison of their retention times and UV-Vis spectra with those of the standards. The purity of each peak was checked to exclude any contribution from interfering peaks. The concentration of each phenolic compound identified in the extracts was obtained from the calibration curve, which was constructed using a mixture of the abovementioned standards in a concentration range of between 1 and 50 mg/L in methanol:water (50:50). The results were expressed in milligrams of compound per liter (mg/L).

## 3. Results

### 3.1. Palynological Analysis

**Figure 1:**
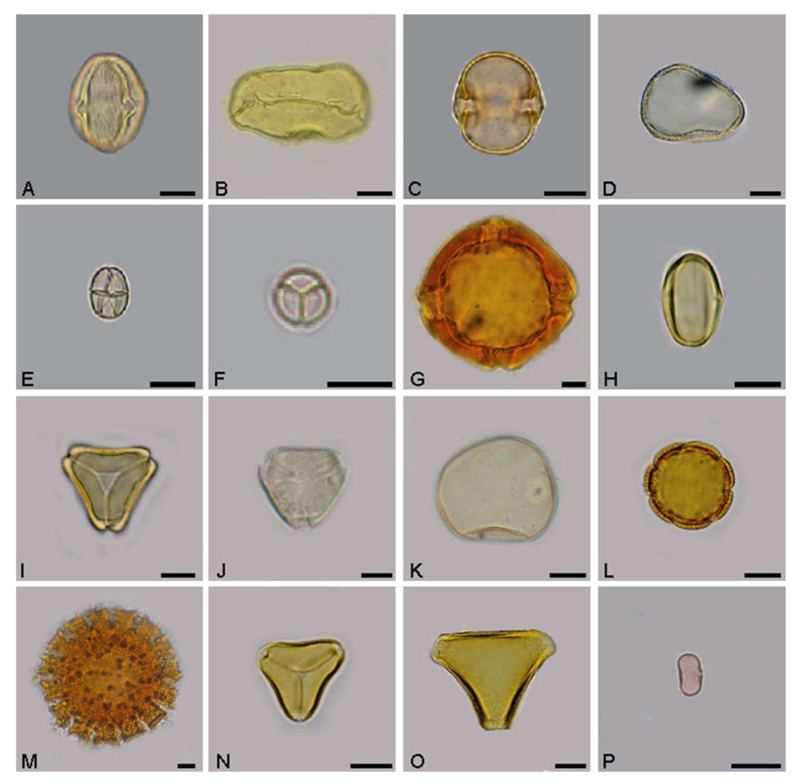
Pollen types found in geopropolis samples collected in an Atlantic Forest area in the state of Bahia (Northeast Brazil). (**A**) Anacardiaceae: *Schinus terebinthifolius*. (**B**) Arecaceae: *Syagrus coronata*. (**C**) Burseraceae: *Protium heptaphyllum*. (**D**) Cyperaceae: *Rhynchospora cephalotes*. (**E**,**F**) Fabaceae: (**E**) *Mimosa pudica*; (**F**) *Mimosa tenuiflora.* (**G**) Malpighiaceae: *Stigmaphyllon blanchetii*. (**H**) Melastomataceae: *Miconia*. (**I**,**J**) Myrtaceae: (**I**) *Eucalyptus*; (**J**) *Myrcia* I. (**K**) *Poaceae*. (**L**,**M**) Rubiaceae: (**L**) *Borreria verticillata*; (**M**) *Richardia grandiflora*. (**N**,**O**) *Sapindaceae:* (**N**) *Cupania*; (**O**) *Serjania* I. (**P**) Urticaceae: *Cecropia*. (Bar = 10 μm).

In the samples analyzed, 75 pollen types were found and 59, belonging to 28 botanical families, were properly identified. The *Fabaceae* family was the most prevalent, followed by the *Myrtaceae* family with seven types. The *Rubiaceae* family also had a great representation, with the presence of five types in total (Figure 1). The types *Cecropia* (*Urticaceae*, Figure 1P), *Eucalyptus* (*Myrtaceae*, Figure 1I), *Mimosa pudica* (*Fabaceae*, Figure 1F), and *Myrcia I* (*Myrtaceae*, Figure 1J) were present in all samples analyzed. The types *Protium heptaphyllum* (*Buseraceae*, Figure 1C) and *Schinus terebinthifolius* (*Anacardiaceae*, Figure 1A), both considered sources of resin for bees, were obtained—56.25% and 81.25% of distribution frequency, respectively. In addition, nine other types, *Borreria verticillata* (Figure 1L), *Miconia* (Figure 1H), *Mimosa tenuiflora* (Figure 1E), *Myrcia II*, *Poaceae* (Figure 1K), *Rhynchospora cepholotes* (Figure 1D), Senna, *Serjania* (Figure 1O), and *Solanum paniculatum,* were also classified as very frequent as they had distribution frequency values greater than 50%.

Overall, the geopropolis from the rainy season presented 53 pollen types, and that from the dry season, 45 types. There were 38 common pollen types: *Schinus terebinthifolius*, *Mimosa pudica*, *Mimosa tenuiflora*, *Miconia-Melastomataceae*, *Eucalyptus*, *Myrcia I*, *Poaceae*, and *Cecropia*.

### 3.2. Physicochemical Analysis

Table 1 summarizes the results obtained for the physicochemical analyses from the geopropolis samples collected in the rainy and dry seasons. Regarding the values obtained for pH (acidic samples) ash, moisture, and waxes, these were identical in the samplings carried out in both seasons. The electrical conductivity and total phenols and flavonoids of the geopropolis samples collected in the rainy season had the highest values and were statistically different from those quantified in the dry season (Tukey’s test). This result can be explained by its chemical variability as a result of edaphoclimatic differences that affect the resin foraging and pollen profile [27,28].

### 3.3. Determination of Phenols

Table 2 shows the HPLC quantified values for the phenols of the geopropolis samples collected in the wet and dry seasons. The composition of geopropolis in phenolic compounds assessed using HPLC is abundant and diverse (Table 2). In our samples, hydroxybenzoic acids, flavonoids, citrus flavonoids, and carboxylic acids were quantified. The HPLC chromatogram displaying the mix of standard phenolic compounds, along with the chromatograms obtained for the rainy geopropolis propolis sample and the dry geopropolis sample, are available in the supplementary material, specifically in Figures S1 and S2, respectively It was found that for most compounds, the amount observed in the sample collected in the rainy season was almost double that quantified in the dry season.

It was also found that gallic acid, protocatechuic acid (hydroxy benzoic acids), catechin (flavonoids), and naringin (citrus flavonoid) were present in both samples analyzed. Quercetin, rutin, and caffeic acid were not observed in any of the samples analyzed. Of the compounds mentioned, naringin stands out, whose content in the geopropolis samples obtained in the rainy season (45.39 mg/L) was 1.5 times higher than the values observed in the dry season samples (29.87 mg/) (Table 2).

### 3.4. Biological Activities

The results obtained for the antioxidant capacity, hyaluronidase enzyme inhibition, and acetylcholinesterase inhibition induced by geopropolis extracts are summarized in Table 3.

#### 3.4.1. Antioxidant Capacity

Regarding antioxidant capacity, the observed values ranged from IC_50_ = 0.058 mg/mL (DPPH) to 89.52% (β-carotene). It should be noted that the higher the CI values_50_ were, the lower the antioxidant capacity was. The geopropolis collected in the rainy season showed a higher antioxidant capacity than the samples obtained in the dry season. The antioxidant capacity assessed using both the DPPH and reducing power methods was approximately three and a half times lower than that obtained for the standard (ascorbic acid). There were significant differences in antioxidant capacity between the samples when analyzed using the different methods, apart from DPPH. For both samples, the method used to evaluate the antioxidant capacity overall significantly influenced the results obtained.

#### 3.4.2. Anti-Inflammatory Activity

The inhibition of hyaluronidase enzyme activity allows the indirect assessment of the effect of geopropolis extracts on anti-inflammatory activity.

It was found that the anti-inflammatory activity induced by geopropolis collected in the dry season was lower (26.62 ± 0.90%), with significant differences observed compared to that of the rainy season (30.95 ± 1.28%).

#### 3.4.3. Inhibition of Acetylcholinesterase

The link between acetylcholinesterase and Alzheimer’s disease results from decreased levels of acetylcholine in the brain. In this study, we tested the effect of geopropolis extracts on this enzyme. The concentrations of extract required to cause a 50% inhibition of the activity of this enzyme were found to be 0.281 ± 0.01 and 0.3542 ± 0.0006 µg/mL for wet and dry season geopropolis, respectively, with statistically significant differences between the effects induced by the two samples. Compared to the geopropolis extract, eserine (control) had a more pronounced effect on the inhibition of this enzyme.

#### 3.4.4. Antimicrobial Activity

From the analysis in Table 4, it was found that all the extracts of the samples analyzed showed antimicrobial activity against Gram-positive and Gram-negative bacteria and yeasts (Table 4). Overall, *Staphylococcus aureus* ESA 321, a clinical isolate from pus (MIC = 0.05 ± 0.02 and MIC = 0.12 ± 0.01 mg/mL for the rainy and dry seasons, respectively), was the most sensitive microorganism to the action of both samples under study, and *Candida albicans* ESA115, isolated from a sputum (MIC = 2.5 ± 0.11 and MIC = 5.00 ± 0.01 mg/mL for the rainy and dry seasons, respectively), was the most resistant. For *Proteus mirabilis*, MIC values were between 0.7 and 1.0 mg/mL [29].

Regarding the minimum bactericidal concentration (MBC), the following values were obtained for *S. aureus:* rainy season—2.25 ± 0.01 mg/mL and 1.75 ± 0.01 mg/mL for the dry season. The values observed for this parameter in *C. albicans* were 5.20 ± 0.02 mg/mL and 5.00 ± 0.04 mg/mL for the rainy and dry seasons, respectively. Overall, the inhibition induced on the various microorganisms under study by the samples collected in the rainy season was higher than that induced by the samples obtained in the dry season.

The difference in the efficiency of propolis on the control of Gram-positive and Gram-negative bacteria may be due to the difference in the structure of the cell wall, wherein the Gram-negative ones are more complex, preventing the action of compounds with an antimicrobial effect. Note that this behavior was observed in both samples tested [11].

#### 3.4.5. Antimutagenic Activity

Table 5 shows the results obtained for the antimutagenic activity induced by geopropolis extracts. All samples were found to have anti-genotoxic action, with values ranging from 23.01 ± 2.05% to 88.59 ± 1.71%; in fact, both extracts decreased gene conversion frequencies (Table 5). The percentage-of-survival results showed a significant interaction term between the dry/rainy geopropolis samples (factor 1) and geopropolis extract amount (factor 2) (*p*-value = 0.031) in the two-way ANOVA model (R^2^ = 0.914; *p*-value < 0.001; residual standard error = 4.70). Geopropolis extract, regardless of harvest time, was found to reduce the survival rate of *S. cerevisiae* D7 (ATCC 201137), an effect that became more pronounced as the concentration of extract added increased.

When *S. cerevisiae* cells were incubated in the presence of geopropolis and EMS (ethyl methanesulfonate; mutagen), both samples were found to have anti-genotoxic action, causing a reduction in both mutant colonies and gene conversion.

The dry geopropolis had higher gene-conversion and colony values compared to rainy geopropolis. Furthermore, the increase in geopropolis extract concentration from 2.5 to 5.0 mg/mL significantly decreased the colony values (two-way ANOVA; R^2^ = 0.893; *p*-value < 0.001; residual standard error = 3.14; interaction term *p*-value = 0.148). As for the mutant colony results, the interaction term was significant (*p*-value = 0.018) in the two-way ANOVA model (R^2^ = 0.960; *p*-value < 0.001; residual standard error = 9.45). The results showed that as the geopropolis-extract concentration increased, the effects on mutant colonies appeared to converge for the two samples.

## 4. Discussion

### 4.1. Palynological Analysis

In this study, where two samples of geopropolis from the Atlantic biome were evaluated, 75 different pollen types were detected. This was much more than the number detected by other studies, for example, the study performed by Barros et al. [30], who analyzed 16 samples of geopropolis produced in a resting area in the state of Maranhão and only identified 38 pollen types. These results reveal the diversity of plant taxa available in this region as sources for *Mellipona scutellaris* throughout the year. Among these various types, *Eucalyptus* (*Myrtaceae*) emerges as particularly significant due to its widespread presence and distribution. A study conducted by Barth et al. [31] in Southeastern Brazil, focusing on geopropolis, classified the Eucalyptus type as the dominant pollen source, accounting for more than 45% of the analyzed samples. Notably, plant species associated with this type frequently appear in research related to propolis and other beekeeping/melipon products in Brazil.

Despite not being a native species to the Brazilian flora, Eucalyptus has demonstrated remarkable adaptability to the country’s climate and soil conditions. Since the 1990s, it has been actively utilized in an extractive manner across various regions of Brazil, as documented by Araújo et al. [32]. Resin constitutes a noteworthy component of geopropolis, yet the factors influencing bees’ preferences for specific resin sources remain a mystery, even though they exhibit discerning choices in this regard [30]. Stingless bee species exhibit regional variations in collecting resins from different plants, making the presence of their pollen in geopropolis a valuable clue for potential resin sources [33]. Notably, *Schinus terebinthifolius* (Anacardiaceae) and *Cecropia* (Urticaceae) species, as identified by [13], are potential resin providers for Apis mellifera propolis in the North Coast region of Bahia, coinciding with the study area. Pollen referring to these species was very frequent in the analyzed samples and may also be indicative of sources of resin used by *M. scutellaris* to produce geopropolis in this region.

### 4.2. Physicochemical Analysis

The composition of geopropolis is complex as it contains mineral particles or ash from the soil. These particles are incorporated into this product when bees encounter the soil during the collection of resins and other materials used in its production. Geopropolis is mainly known for its pharmacological and therapeutic properties. Despite the great multiplicity of bee species capable of producing this product, studies on its physical–chemical characteristics and biological properties are recent, and some of them scarce or non-existent. In this context, delving into research concerning the chemical composition and biological activity of stingless bee geopropolis holds pivotal significance. Such investigations not only aim to establish a quality benchmark but also seek to unravel the mechanisms of action inherent to this remarkable natural product.

According to the literature, geopropolis is generally slightly acidic, with pH values ranging between 3.5 and 5.5. Phenolic acids and flavonoids, which are relevant in the chemical composition of geopropolis, are the main factors responsible for this characteristic [1]. However, it is important to note that pH can vary with the origin of geopropolis and even within the same bee colony. Although this parameter has not been contemplated in the legislation, the values obtained in our study corroborate those described by da Silva Cruz et al. [2]. According to this researcher, pH is one of the main factors responsible for the stability of this product, as it influences the multiplication of its microbiota.

Specific studies on the electrical conductivity of geopropolis are insufficient or non-existent. However, it is possible to infer that the conductivity is probably low, particularly due to the high content of waxes and resins that are part of its composition.

Regarding ashes, Oliveira et al. [34] analyzed geopropolis from different stingless bee species, reporting values ranging from 75.40 ± 0.23% to 85.43 ± 0.53% (m/m), which corroborate the results obtained in this study. According to Pereira et al. [35], the high values of this parameter compared to those observed in other bee products are justified by the fact that geopropolis is a mixture of clay/earth and resin, and consequently, its content of minerals and trace elements is higher. However, it is important to note that the concentration and specific composition of minerals in ash can vary depending on the region and the plants used by bees [36]. Sawaya et al. [33] reported that high ash contents indicate that the product has not been adulterated.

Concerning moisture, the values obtained in our study were identical to those reported by Araújo et al. [37] for *Melipona scutellaris* and *M. fasciculata* species (1.40% to 1.76%), suggesting that the moisture content of stingless bees is low. Indeed, da Silva Cruz et al. [2] found that the moisture content of geopropolis was six and eleven times lower than those of honey and pollen produced by *M. scutellaris*, respectively.

The waxes in geopropolis are important components of the structures of hives, also possessing therapeutic properties. The results obtained in this study are identical to those observed by Araújo et al. [37] in geopropolis of *M. scutellaris* (4.33%). However, they are higher than those described by the same author in geopropolis of *M. fasciculata* (0.95%) and by Cardozo et al. [38] in geopropolis of *M. Jataí*, *M. Mandaçaia*, and *M. Mandurí*. Araújo et al. [39] evaluated propolis collected in the same apiary and obtained values for this parameter that ranged between 25.45 and 24.09%, demonstrating that the product produced by *Apismellifera* had higher values than the geopropolis of stingless bees. These differences can be justified by the fact that the chemical composition of geopropolis varies with the flora and the geographical region where it is collected [39].

Phenolic compounds are present in several natural products, namely geopropolis, and are responsible for many of the biological properties associated with these products. However, it is important to note that the quantities, qualities, and beneficial effects of these substances depend on the specific chemical composition of each sample and the concentration of compounds present [40,41]. In samples of geopropolis from *Melipona fasciculata* collected by Dutra et al. [42] in the savannah of Maranhão state, the values of this parameter ranged from 14.14 to 67.46%. These values were higher than those observed by these authors in the geopropolis of *M. scutellaris* obtained in the state of Bahia (Brazil). According to Araújo et al. [39], the flora of the region and the species of stingless bee can quantitatively influence the content of total phenols in this bee product.

Regarding flavonoids, Cardozo et al. [38] obtained lower contents (0.66 to 0.02%) than those quantified in our study. This variation in flavonoid content between different geopropolis samples has already been reported by other researchers, for example, ref. [42], who evaluated the flavonoid content in geopropolis produced in Maranhão and obtained values between 0.17 and 6.0%. Sawaya et al. [33] observed flavonoid contents in extracts of geopropolis of *Melipona fasciculata* from the same state ranging from 0.85% to 1.85%. According to these researchers, the variations in the content of flavonoid compounds in the different studies may result from differences in the time and time of collection of the geopropolis, vegetation, or the type of soil collected by the bees, since the extraction methods and the extracting solvents used were the same.

### 4.3. Quantification of Phenolic Compounds

According to Righi et al. [43], when dealing with complex mixtures, chromatographic methods are essential for assessing active compounds. In our study, we noted variations in phenolic compound levels between samples collected in different seasons. Naringin, the most abundant compound in both samples, was about 1.5 times lower in concentration during the dry season compared to the rainy season. Kasote et al. [44] found a similar phenolic compound profile in geopropolis from various Indian states, with naringin being the predominant compound. In contrast, gallic acid was a primary component in propolis samples from stingless bees collected in Brazilian states like Pernambuco, Parana, and São Paulo [45]. Santos et al. [6] also reported that the hydroalcoholic extracts of geopropolis from *Melipona orbignyi* stood out for presenting flavonoids, aromadendrin, and naringenin. Naringin is a glycosylated flavanone commonly found in citrus fruits and grapes. This compound possesses antioxidant, anti-inflammatory, antimutagenic, and antimicrobial properties [46,47].

### 4.4. Antioxidant Activity

According to the literature, antioxidant activity depends on the chemical composition of geopropolis, particularly the quantity and quality of phenols and flavonoids [48]. Free radical scavenging is one of the methodologies frequently used to assess antioxidant capacity [20]. DPPH free radical scavenging measures the ability of the analyzed substances to donate hydrogen ions [49]. The reducing power of a given product indicates its antioxidant potential [20]. The β-carotene/linoleic acid system measures the antioxidant potential of a substance as the ability to sequester the free radical generated by the peroxidation of linoleic acid [50].

Santos et al. [6], when analyzing the antioxidant activity of geopropolis from *Melipona orbignyi* using the DPPH method, obtained IC50 values of 18.3 µg/mL. These values were lower than those determined in our study, suggesting that this biological property is influenced by the local flora and bee species. However, Araújo et al. [39], in their investigation of geopropolis from *Melipona scutellaris* collected in an area with vegetation identical to that in our study, reported IC_50_ values determined by the DPPH method that were remarkably similar to those observed in our work. The values obtained by Batista et al. [51] in hydro-ethanolic extracts of geopropolis produced by *M. fasciculata* in Maranhão also corroborate our determinations. Even though our samples had the same geographical origin, differences were also observed regarding the botanical origin and the amount and type of phenolic compounds.

### 4.5. Anti-Inflammatory Activity

The enzyme hyaluronidase breaks down hyaluronic acid, a key component found in the extracellular matrix of connective tissue. The assessment of anti-inflammatory activity based on this enzyme is used to determine its ability to reduce inflammation and modulate the inflammatory response. These assays are important to understand the mechanisms of action of hyaluronidase and may pave the way for the development of new therapeutic approaches in the treatment of inflammatory diseases [23]. Its activity is relevant in physiological and pathological processes, and it is also used as a therapeutic adjuvant in several clinical contexts. The effect of hyaluronidase enzyme inhibition induced by geopropolis used in our study was lower than that reported by Santos et al. [6] in geopropolis collected in Mato Grosso do Sul, Brazil.

### 4.6. Inhibition of Acetylcholinesterase

Acetylcholinesterase (AChE) is vital for breaking down acetylcholine, a neurotransmitter involved in nerve signaling. Compounds that inhibit or reactivate AChE have potential uses in Alzheimer’s disease and organophosphate poisoning treatment [37]. In our study, there was a greater inhibition of this enzyme by the sample collected in the rainy season. This fact may be associated with the higher content of phenols present in this sample; however, further studies involving larger numbers of samples are needed to clarify the effect of this bee product, produced almost exclusively by stingless bees, on acetylcholinesterase, as we are not aware of other studies carried out in this area.

### 4.7. Antimicrobial Activity

In this study, all samples showed antimicrobial activity against Gram-positive and Gram-negative bacteria and yeasts. Overall, *Staphylococcus aureus* ESA 321, a clinical isolate from pus, was the most sensitive microorganism to the action of both matrices under study, and *Candida albicans* ESA115, isolated from a sputum, was the most resistant. The geopropolis extract showed greater efficacy against *Proteus mirabilis* than previously reported by Campos et al. [29] in a study involving a geopropolis extract produced by stingless bees (*Tetragonisca fiebrigi*) in Grandes Dourados. These authors observed MIC values for this bacterium of between 2.25 and 3.08 mg/mL, i.e., higher than those obtained in our study. The fact that yeasts are eukaryotic organisms may justify their behavior towards bacteria. The higher resistance of Gram-negative bacteria can be explained by their more complex cell wall composition, which makes it more difficult for antimicrobials to enter. In all cases, the resistance of the reference microorganisms was higher than that of the clinical isolates, probably because the clinical isolates had already acquired antimicrobial resistance. Our results are identical to those reported by Santos et al. [6] in geopropolis produced in Grandes Dourados by *Melipona orbignyi*.

Liberio et al. [52] found that geopropolis samples produced by *M. fasciculata* inhibited the growth of *C. albicans*. Da Cunha et al. [53] studied the antimicrobial effect of geopropolis samples of *M. scutellaris*, collected in the study area where the samples used in our study were from, against Gram-positive and Gram-negative bacteria and found that the growth of *S. aureus* was inhibited for concentrations higher than 50 μg/mL while the MIC for *Pseudomonas aeruginosa* was higher than 1600 μg/mL. These observations corroborate our results, indicating that Gram-positive bacteria are more sensitive than Gram-negative bacteria. Valcanaia et al. [54] reported that natural products showing antimicrobial activity with MIC (minimum inhibitory concentration) values below 100 µg/mL can be considered good antimicrobial agents. In this context, the results obtained in this work suggest that geopropolis can be used as an antibacterial agent, with its antifungal effect being more limited. 

According to what has been reported in the literature, phenolic compounds are said to be mainly responsible for the biological properties of hive products, particularly geopropolis. Polyphenol derivatives common in propolis have been shown to exert a wealth of therapeutic benefits [55], having antioxidant capacity (can interrupt the chain reactions caused by free radicals but can also inhibit their formation), antimicrobial (can induce the permeabilization of the microbial cytoplasmic membrane and inhibition of the synthesis of nucleic acids in Gram-negative/Gram-positive bacteria and synthesis of ATP and interruption of electron transport) and anti-inflammatory activity and pro-inflammatory enzymes [6] such as nitric oxide synthase and cyclooxygenase-2 [56].

### 4.8. Antimutagenic Activity

The use of the yeast *Saccharomyces cerevisiae* ATCC 20113–D7 in the evaluation of antimutagenic activity involves the analysis of the effect of geopropolis in preventing or reducing genetic mutations induced by mutagens. In the present study, three concentrations of geopropolis were used: 0.0, 0.25, and 0.5, with methyl methanesulfonate (EMS) at a concentration of 0.5%. EMS is an alkylation agent that produces random mutations in genetic material via nucleotide substitution. Geopropolis, depending on the concentration, caused a decrease in the frequency of gene conversion and the number of mutant colonies induced by the EMS mutagen.

## 5. Conclusions

The analysis of the geopropolis samples gathered within the Atlantic Forest of Bahia revealed that *Schinus terebinthifolius* and *Cecopia* species served as the resin providers utilized by *Melipona scutellaris* bees to produce this product. In this study, for the first time, we demonstrated that the timing of harvest influences the chemical profile and bioactivity of *M. scutellaris* geopropolis. Antioxidant, antibacterial, antimutagenic, anti-cholinesterase, and inflammatory capabilities, particularly evident in the samples obtained during the rainy season, suggest the potential of this product for preventing inflammatory, oxidative, and infectious diseases. Although the meliponary under study is in an area where extractive agriculture is practiced, the maintenance of the vegetation ensures resource availability throughout the year. However, it is necessary to protect these species to ensure that the production of this natural product becomes a sustainable source of income. Additionally, these findings show the importance of studies that search for effective alternative therapies in facing the global increase in resistance to antibiotics currently used and point out the extracts as possible candidates for the development of new treatments against bacterial infections. On the other hand, the high variability of the chemical profile of geopropolis of *Melipona* species is a factor requiring strategies aiming at the standardization of the product.

## Figures and Tables

**Table 1:** Physicochemical characteristics of geopropolis samples produced in an Atlantic Forest area, Bahia State, Brazil (mean and standard deviation).

ASamples	pH	Conductivity (mS/cm)	Moisture (%)	Ash (%)	Waxes (%)	Phenolic Compounds (%)	Flavonoid Compounds (%)
Rainy geopropolis (summer) *	4.110 ± 0.060 a	36.000 ± 0.000 a	2.630 ± 0.070 a	76.130 ± 0.860 a	3.370 ± 0.290 a	25.130 ± 0.140 a	3.920 ± 0.090 a
Dry geopropolis (winter) *	4.200 ± 0.040 a	34.000 ± 0.820 b	2.660 ± 0.020 a	76.340 ± 0.070 a	3.170 ± 0.720 a	19.30 ± 0.150 b	2.090 ± 0.370 b

* Different letters (a, b) indicate significant differences (Tukey’s test).

**Table 2:** Phenolic compounds identified and quantified in propolis and geopropolis extracts using HPLC-UV; results are expressed in mg/L (ND: not detected; LOQ: limit of quantification; LOD: limit of detention).

Compound	Retention Time (min)	Rainy Geopropolis	Dry Geopropolis
gallic acid	7.14	5.820	5.840
protocatechuic acid	12.74	2.530	2.460
Catechin	18.30	8.390	2.640
vanillic acid	22.79	2.430	ND
caffeic acid	24.40	ND	ND
Epicatechin	25.38	1.720	1.320
p-coumaric acid	31.48	<LOQ	<LOD
ferulic acid	34.38	ND	ND
Naringin	42.17	45.390	29.870
Rutin	45.56	ND	ND
cinnamic acid	49.71	2.540	<LOD
Naringenin	55.25	2.110	<LOQ
Quercetin	54.07	ND	ND

**Table 3:** Values obtained for antioxidant capacity, hyaluronidase enzyme inhibition, and acetylcholinesterase inhibition in geopropolis samples, collected in the rainy and dry seasons.

	Scavenging of DPPH Radicals—IC50 (mg/mL)	Reducing Power—IC50 (mg/mL)	β-Carotene/Linoleic Acid (%)	Hyaluronidase (30 mg/mL) (%)	Acetylcholinesterase—IC50 (µg/mL)
Rainy Geopropolis *	0.058 ± 0.005 a	0.310 ± 0.008 a	89.52 ± 0.57 a	30.95 ± 1.28 a	0.28 ± 0.01 b
Dry Geopropolis *	0.064 ± 0.004 a	0.220 ± 0.001 b	75.54 ± 1.10 b	26.62 ± 0.90 b	0.354 ± 0.006 a
Ascorbic acid	0.015 ± 0.001	0.074 ± 0.013	-	-	-
BHA	-	-	92.450 ± 0.001	-	
Eserine					0.005 ± 0.001

* Different letters (a, b) indicate significant differences (Tukey’s test).

**Table 4:** Minimum Inhibitory Concentration (MIC) and Minimum Bactericidal Concentration (MBC) of geopropolis extracts, gentamicin, and amphoterecin against isolated (ESA) and reference (ATCC) microorganisms (*—not applicable to the microorganism).

Microorganisms	Rainy Geopropolis (mg/mL)		Dry Geopropolis (mg/mL)		Gentamicin (mg/mL)	Amphoterecin (mg/mL)
	MIC	MBC	MIC	MBC		
Gram-positive bacteria						
*Staphylococcus aureus* ATCC 4330	0.05 ± 0.12	2.01 ± 0.07	0.10 ± 0.02	1.25 ± 0.06	0.03 ± 0.01	*
*Staphylococcus aureus* ESA 321	0.05 ± 0.02	2.25 ± 0.01	0.12 ± 0.01	1.75 ± 0.01	0.13 ± 0.02	*
Gram-negative bacteria						
*Proteus mirabilis* ATCC 25933	1.00 ± 0.03	1.50 ± 0.03	0.50 ± 0.15	1.34 ± 0.04	0.06 ± 0.01	*
*Proteus mirabilis* ESA 229	0.07 ± 0.01	1.76 ± 0.07	0.10 ± 0.05	1.12 ± 0.01	0.005 ± 0.001	*
Fungi						
*Candida albicans* ATCC 10231	1.35 ± 0.01	5.01 ± 0.17	2.5 ± 0.04	5.00 ± 0.06	*	0.002 ± 0.001
*Candida albicans* ESA 115	2.50 ± 0.11	5.22 ± 0.02	5.00 ± 0.01	5.00 ± 0.04	*	0.021 ± 0.008

**Table 5:** Effects of the hydroethanolic extract of geopropolis of Me on the survival percentage of yeast cells (*Saccharomyces cerevisiae*) (diploid line D7 ATCC 201137), conversion of genes, and mutant colonies.

Sample	Treatment		Survivals (%)	Gene Conversion Colonies/10^5^	Mutant Colonies/10^6^
	[Extract] (mg/mL)	EMS (mg/mL)			
Dry geopropolis	0	5.0	88.18 ± 1.60	51.55 ± 0.60 a	380.56 ± 7.67
	2.5	5.0	36.24 ± 2.72	37.33 ± 1.27 b	323.07 ± 1.36
	5.0	5.0	28.84 ± 2.19	31.07 ± 0.98 c	302.58 ± 3.43
Rainy geopropolis	0	5.0	88.59 ± 1.71	51.34 ± 0.63 d	404.04 ± 9.46
	2.5	5.0	30.64 ± 2.38	43.82 ± 3.95 e	354.95 ± 17.78
	5.0	5.0	23.01 ± 2.05	37.21 ± 1.87 f	311.88 ± 1.52

Different letters (a, b, c, d, e, f) indicate significant differences (multiple comparisons using Tukey’s HSD).

## Supplementary Materials

The following supporting information can be downloaded at: https://www.mdpi.com/article/10.3390/microorganisms11112779/s1, Figure S1: HPLC chromatogram obtained for the rainy geopropolis propolis sample at 280 nm; Figure S2: HPLC chromatogram obtained for the dry geopropolis sample at 280 nm; Table S1: Phenolic compounds analysed by HPLC.
