# Supplementary material for: Melipona scutellaris Geopropolis: Chemical Composition and Bioactivity
Source: Microorganisms. 2023 Nov 15;11(11):2779. doi: 10.3390/microorganisms11112779 (PMC10673356; doi:10.3390/microorganisms11112779)
Supplement: Supplementary file 1 [file microorganisms-11-02779-s001.zip › microorganisms-2638508-supplementary.pdf]

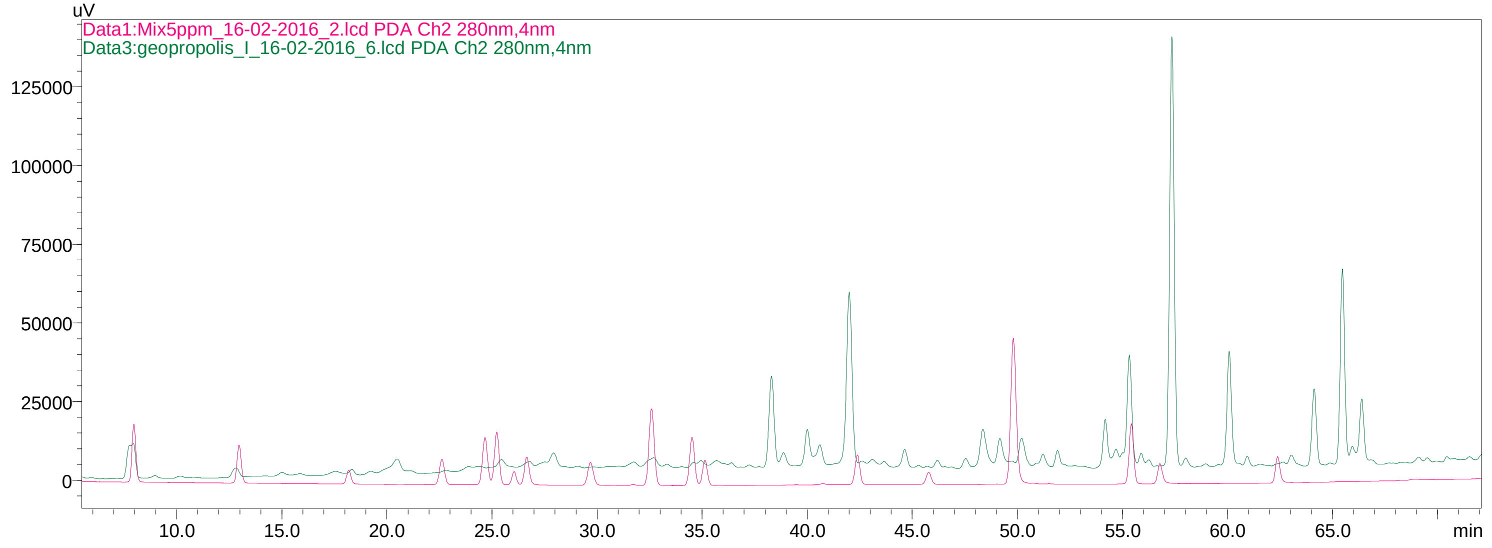

Figure S1. HPLC chromatogram obtained for the rainy geopropolis propolis sample at 280 nm.

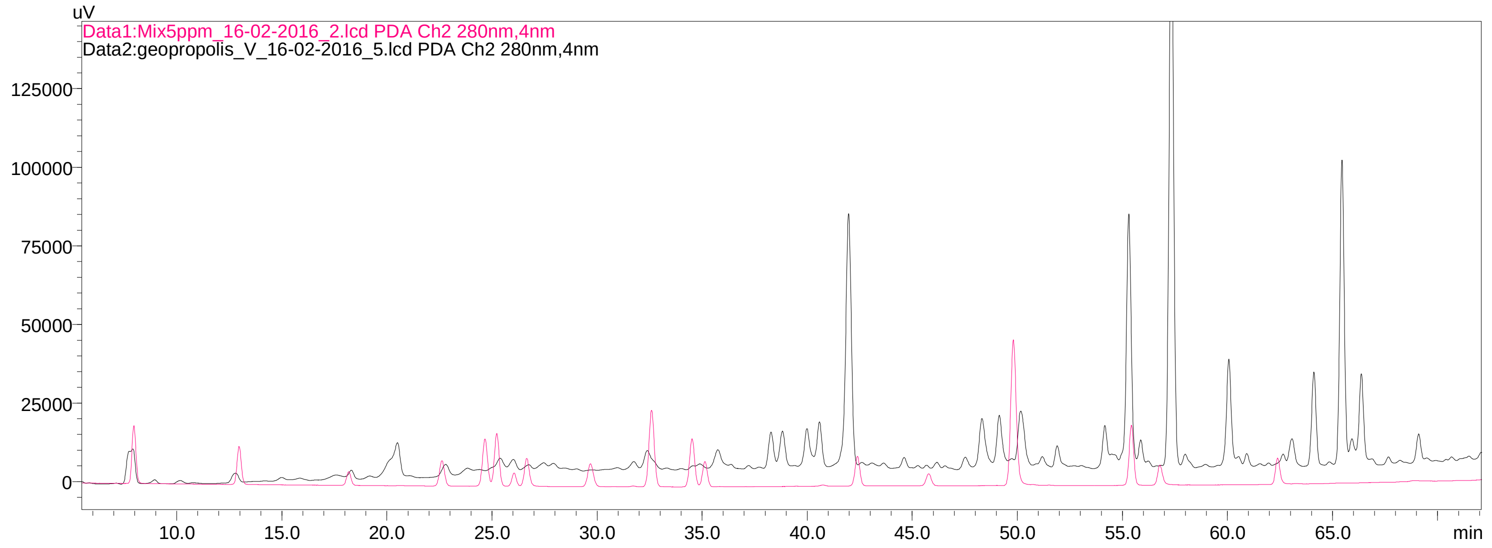

Figure S2. HPLC chromatogram obtained for the dry geopropolis sample at 280 nm.

Table S1. Phenolic compounds analysed by HPLC.

Compound	Retention time (min)	Dry geopropolis (mg/L)	Rainy geopropolis (mg/L)
gallic acid	7,137	5.84	5.82
protocatechuic acid	12,743	2.46	2.53
catechin	18,297	2.64	8.39
vanillic acid	22,788	ND	2.43
caffeic acid	24,395	ND	ND
epicatechin	25,376	1.32	1.72
p-coumaric acid	31,481	<LOD	<LOQ
ferulic acid	34,376	ND	ND
naringin	42,166	29.87	45.39
Rutin	45,556	ND	ND
cinnamic acid	49,709	<LOD	2.54
Naringenin	55,251	<LOQ	2.11
quercetin	54,071	ND	ND

ND – not detected.
